# The impact of dialysis therapy on older patients with advanced chronic kidney disease: a nationwide population-based study

**DOI:** 10.1186/s12916-014-0169-3

**Published:** 2014-10-06

**Authors:** Chia-Jen Shih, Yung-Tai Chen, Shuo-Ming Ou, Wu-Chang Yang, Shu-Chen Kuo, Der-Cherng Tarng, for the Taiwan Geriatric Kidney Disease Research (TGKD) Group

**Affiliations:** Department of Medicine, Taipei Veterans General Hospital, Yuanshan Branch, Yilan, Taiwan; School of Medicine, National Yang-Ming University, Taipei, Taiwan; Department of Medicine, Taipei City Hospital Heping Fuyou Branch, Taipei, Taiwan; Division of Nephrology, Department of Medicine, Taipei Veterans General Hospital, No. 201, Section 2, Shih-Pai Road, Taipei, 11217 Taiwan; Institutes of Physiology and Clinical Medicine, National Yang-Ming University, Taipei, Taiwan; National Institute of Infectious Diseases and Vaccinology, National Health Research Institutes, Miaoli County, Taiwan; Division of Infectious Diseases, Taipei Veterans General Hospital, Taipei, Taiwan

**Keywords:** Advanced chronic kidney disease, Dialysis, Conservative care, Older people

## Abstract

**Background:**

Older patients with advanced chronic kidney disease (CKD) face the decision of whether to undergo dialysis. Currently available data on this issue are limited because they were generated by small, short-term studies with statistical drawbacks. Further research is urgently needed to provide objective information for dialysis decision making in older patients with advanced CKD.

**Methods:**

This nationwide population-based cohort study was conducted using Taiwan’s National Health Insurance Research Database. Data from 2000 to 2010 were extracted. A total of 8,341 patients ≥70 years old with advanced CKD and serum creatinine levels >6 mg/dl, who had been treated with erythropoiesis-stimulating agents were included. Cox proportional hazard models in which initiation of chronic dialysis was defined as the time-dependent covariate were used to calculate adjusted hazard ratios for mortality. The endpoint was all-cause mortality.

**Results:**

During a median follow-up period of 2.7 years, 6,292 (75.4%) older patients chose dialysis therapy and 2,049 (24.6%) received conservative care. Dialysis was initiated to treat kidney failure a median of 6.4 months after enrollment. Dialysis was associated with a 1.4-fold increased risk of mortality compared with conservative care (adjusted hazard ratio 1.39, 95% confidence interval 1.30 to 1.49). In subgroup analyses, the risk of mortality remained consistently increased, independent of age, sex and comorbidities.

**Conclusions:**

In older patients, dialysis may be associated with increased mortality risk and healthcare cost compared with conservative care. For patients who are ≥70 years old with advanced CKD, decision making about whether to undergo dialysis should be weighted by consideration of risks and benefits.

**Electronic supplementary material:**

The online version of this article (doi:10.1186/s12916-014-0169-3) contains supplementary material, which is available to authorized users.

## Background

Older patients with chronic kidney disease (CKD) stage 5 comprise a rapidly growing emerging population that may face the dilemma of whether to undergo dialysis or receive conservative care [1]. Although patients with end-stage renal disease (ESRD) tend to choose dialysis therapy over conservative care, the use of dialysis to treat kidney failure peaks in patients who are 75 years old and declines thereafter [2,3]. The perceived financial burden of dialysis, as well as a high comorbidity rate, uncertainty about the treatment’s long-term benefit, and sense of life completion and acceptance of death, leads many older patients to forego dialysis [4]. The most critical concern of older patients with CKD stage 5 is whether dialysis provides benefits such as increased life expectancy and improved functional ability at the end of life [5,6]. The new guidelines of the Renal Physicians Association and the American Society of Nephrology on the initiation and withholding or withdrawal of dialysis emphasize shared decision making with patients, their family members and physicians in charge of their care [7]. Although comprehensive physician–patient communication may help to achieve treatment goals and avoid unnecessary medical expenditure [8,9], independent and objective evidence of the comparative effectiveness of treatment options is still needed to guide older patients’ decision making about dialysis.

To date, very few studies have investigated the risks and benefits of dialysis therapy for older patients with advanced CKD. Previous studies examining this issue found that dialysis conferred a significant but small advantage over conservative care in older patients [10-12]. However, the statistical power of these studies was limited due to small samples, short follow-up periods and limitations of analytical methodologies. Because initiation of chronic dialysis commonly occurs during follow up in patients with advanced CKD, the use of appropriate statistical methods to calculate the fraction of mortality attributable to dialysis is essential. Accordingly, in this nationwide population-based cohort study based on Taiwan’s National Health Insurance Research Database (NHIRD), we utilized the initiation of chronic dialysis as a time-dependent covariate in Cox regression models to assess the real effect of dialysis on older patients with advanced CKD.

## Methods

### Data source

The present study was performed using Taiwan’s NHIRD. Taiwan launched the national health insurance (NHI) program in 1995. This single-payer system covers approximately 99% of residents. In 1999, as part of the NHIRD project, the Bureau of National Health Insurance began to release patient data in electronic form for research purposes. These de-identified secondary data include all registry and claims data, ranging from demographic data to detailed orders from ambulatory and inpatient care. Diseases are coded according to the *International Classification of Diseases, Ninth Revision, Clinical Modification* (ICD-9-CM). The accuracy of diagnoses registered in the NHIRD has been validated for several diseases, including acute kidney injury [13], chronic kidney disease [14-16], acute coronary syndrome [17], ischemic stroke [18] and diabetes [19]. Because the dataset consisted of de-identified secondary data, this study was exempted from full review by the Institutional Review Board of Taipei City Hospital (TCHIRB-1030407-W).

### Study design

This population-based, observational, retrospective cohort study was performed to determine the association between chronic dialysis and mortality in older patients with advanced CKD. We identified all subjects ≥70 years old in 2000 and extracted all relevant data for these subjects for the study period of January 2000 to December 2010. These data included demographic characteristics, diagnosis and procedure codes, drug prescriptions and information about outpatient visits and hospital admissions. We further extracted data from January 1995 to December 1999 to ensure the availability of information for all individuals for at least five years preceding enrollment; this information was used to identify comorbidities.

Among all individuals ≥70 years old in January 2000 in Taiwan, patients with ICD-9-CM codes for CKD (016.0, 042, 095.4, 189, 223, 236.9, 250.4, 271.4, 274.1, 403–404, 440.1, 442.1, 446.21, 447.3, 572.4, 580–589, 590–591, 593, 642.1, 646.2, 753 and 984) and receiving erythropoiesis-stimulating agents (ESAs) were identified. The first date of prescription of ESAs was defined as the index date. We excluded patients with histories of cancer, patients receiving chronic dialysis or kidney transplantation before or during the 30 days after the index date and patients with follow-up periods <30 days. According to NHI reimbursement regulations, patients with CKD, serum creatinine levels >6 mg/dl (approximately equivalent to glomerular filtration rate [GFR] <15 ml/min/1.73 m^2^), and hematocrit <28% should receive ESAs to maintain a target hematocrit level not to exceed 36%. In addition, unlike the relative low prevalence of ESA utilization (less than 20%) among CKD or ESRD patients in the US [20,21], a report from the Taiwan Department of Health indicated that 85% of patients with advanced CKD stage 5 not yet requiring dialysis received ESAs therapy in 2012 [22], possibly due to convenient medical access and minimal financial barrier of health insurance access in Taiwan. The median hematocrit value at the initiation of dialysis was 24.2% (interquartile range 20.6% to 27.5%) in Taiwan [23]. Thus, the selected cohort in our study is most representative of older patients with advanced CKD not yet requiring dialysis in Taiwan.

During the study period, the records of patients receiving chronic dialysis were extracted from the Registry of Catastrophic Illness. For catastrophic illnesses such as ESRD on chronic dialysis, the government registered the confirmed subjects, after strict verification. After successful certification, ESRD patients on chronic dialysis can be exempted from related medical expenses. Therefore, the application of catastrophic illness certificate for ESRD on dialysis required specialist nephrological reviews in supported medical records, examination reports and imaging studies after careful exclusion of the causes of acute renal failure [24].

### Outcome measures

The endpoint was all-cause mortality. All subjects were followed until death or 31 December 2011.

### Potential confounders

Baseline demographic data, including age, sex, economic status and urbanization level of the patients’ places of residence, were collected. Patients’ systemic health status was evaluated using the Charlson Comorbidity Index (CCI). Each increase in the CCI represents a stepwise increase in cumulative mortality, that is, a score of 0 is associated with a 99% 10-year survival rate and a score of 5 is associated with a 34% 10-year survival rate [25]. The effect of primary renal disease on survival rate was also taken into consideration. Data on other comorbidities that affect survival and are not included in the CCI, such as diabetes mellitus, hypertension, dyslipidemia, atrial fibrillation, valvular heart disease, parkinsonism, autoimmune disease and drug abuse, were also extracted. Prescribed medications that could confound mortality, such as antiplatelet agents, warfarin, angiotensin-converting enzyme inhibitors (ACEIs), angiotensin receptor blockers (ARBs), beta blockers, calcium channel blockers, diuretics, nitrate, statins, dipyridamole, steroids, estrogen or progesterone, non-steroidal anti-inflammatory drugs, selective serotonin re-uptake inhibitors, proton pump inhibitors and oral hypoglycemic drugs, were identified.

### Statistical analysis

The baseline characteristics were first analyzed using descriptive statistics and then compared using Pearson *χ*^2^ tests for categorical variables, and the independent *t*-test and Mann–Whitney *U*-test for parametric and nonparametric continuous variables, respectively. Cox regression models were used to calculate hazard ratios (HRs) and 95% confidence intervals (CIs) characterizing the risk of mortality after chronic dialysis. Because no patient was undergoing chronic dialysis at the time of enrollment, this variable was calculated as a time-dependent covariate to ensure that patients were considered at risk only when they were receiving chronic dialysis. The models allowed patients to switch from one exposure group to another and were adjusted for the variables listed in Table 1. Subgroup Cox regression analyses were performed to examine the effects of age, sex, CCI, underlying disease and time cohort on the risk of mortality after chronic dialysis. Interaction effects were examined using the likelihood ratio test.

**Table 1:** **Baseline characteristics of patients**

		**During follow-up period**		
**Parameters**	**All patients**	**Not receiving chronic dialysis**	**Receiving chronic dialysis**	***P***
Number of patients	8,341	2,049	6,292	
Male, number (%)	3,726 (44.7)	955 (46.6)	2,771 (44.0)	0.042
Age, mean (SD), years	79.4 (7.0)	82.0 (6.4)	78.6 (7.1)	<0.001
Follow-up period, mean (SD), days	1,026 (880)	478 (546)	1,205 (894)	<0.001
Day of chronic dialysis initiation, median (IQR)	-	-	192 (90 to 404)	
Age at chronic dialysis initiation, mean (SD), years	-	-	79.4 (7.1)	
Monthly income (US$), number (%)				0.158
Dependent	3,395 (40.7)	831 (40.6)	2,564 (40.8)	
0 to 637	1,933 (23.2)	505 (24.7)	1,428 (22.7)	
637 to 1,400	2,955 (35.4)	703 (34.3)	2,252 (35.8)	
>1,400	58 (0.7)	10 (0.5)	48 (0.8)	
Urbanization level^a^, number (%)				0.150
1	4,210 (50.5)	1,021 (49.8)	3,189 (50.7)	
2	3,236 (38.8)	800 (39.0)	2,436 (38.7)	
3	740 (8.9)	178 (8.7)	562 (8.9)	
4	155 (1.9)	50 (2.4)	105 (1.7)	
Charlson Comorbidity Index^b^, mean (SD)	5.6 (2.1)	5.7 (2.2)	5.5 (2.1)	0.003
Primary renal disease, number (%)				
Diabetes	3,070 (36.8)	721 (35.1)	2,349 (37.3)	0.080
Glomerulonephritis	4,008 (48.0)	984 (48.0)	3,024 (48.0)	0.979
Secondary glomerulonephritis/vasculitis	86 (1.0)	26 (1.2)	60 (0.9)	0.220
Hypertension/large vessel disease	986 (11.8)	273 (13.3)	713 (11.3)	0.015
Cystic/hereditary/congenital disease	615 (7.3)	142 (6.9)	473 (7.5)	0.377
Miscellaneous conditions	186 (2.2)	54 (2.6)	132 (2.0)	0.152
Comorbidities, number (%)				
Diabetes mellitus	4,807 (57.6)	1,152 (56.2)	3,655 (58.1)	0.137
Hypertension	7,807 (93.6)	1,904 (92.9)	5,903 (93.8)	0.151
Dyslipidemia	3,972 (47.6)	890 (43.4)	3,082 (49.0)	<0.001
Atrial fibrillation	579 (6.9)	170 (8.3)	409 (6.5)	0.005
Valvular heart disease	1,698 (20.4)	472 (23.0)	1,226 (19.5)	0.001
Parkinsonism	617 (7.4)	191 (9.3)	426 (6.8)	<0.001
Autoimmune disease	1,190 (14.3)	309 (15.1)	881 (14.0)	0.225
Drug abuse	42 (0.5)	13 (0.6)	29 (0.5)	0.335
Concomitant medications, number (%)				
Antiplatelet agents^c^	1,948 (23.4)	507 (24.7)	1,441 (22.9)	0.087
Warfarin	59 (0.7)	10 (0.5)	49 (0.8)	0.173
ACE inhibitors or ARB	2,716 (32.6)	678 (33.1)	2,038 (32.4)	0.558
Beta blockers	380 (4.6)	83 (4.1)	297 (4.7)	0.207
Calcium channel blockers	4,868 (58.4)	977 (47.7)	3,891 (61.8)	<0.001
Diuretics	4,707 (56.4)	1,098 (53.4)	3,609 (57.4)	0.003
Nitrate	1,626 (19.5)	376 (18.4)	1,250 (19.9)	0.132
Statins	710 (8.5)	146 (7.1)	564 (9.0)	0.010
Dipyridamole	2,107 (25.3)	463 (22.6)	1,644 (26.1)	0.001
Steroids	690 (8.3)	189 (9.2)	501 (8.0)	0.072
Estrogen or progesterone	38 (0.5)	11 (0.5)	27 (0.4)	0.529
Non-steroidal anti-inflammatory drugs	1,248 (15.0)	312 (15.3)	936 (14.9)	0.699
Selective serotonin re-uptake inhibitors	102 (1.2)	28 (1.4)	74 (1.2)	0.496
Proton pump inhibitors	473 (5.7)	157 (7.7)	316 (5.0)	<0.001
Oral hypoglycemic drugs	1,843 (22.1)	414 (20.2)	1,429 (22.7)	0.018

^a^Urbanization levels in Taiwan are divided into four strata according to Taiwan National Health Research Institute publications. Level 1 designates the most urbanized areas and level 4 designates the least urbanized areas. ^b^The Charlson Comorbidity Index (CCI) is used to determine overall systemic health. Each increase in CCI represents a stepwise increase in cumulative mortality_._^c^Including aspirin, clopidogrel, ticlopidine, and cilostazol. ACE, angiotensin-converting enzyme; ARB, angiotensin II receptor blocker; IQR interquartile range; SD, standard deviation.

Because the baseline characteristics of patients receiving chronic dialysis differed substantially from those receiving conservative care, we also performed a propensity score–matched analysis. Propensity scores representing the likelihood of receiving chronic dialysis were calculated using logistic regression analysis, conditional on the baseline covariates listed in Table 1. For each subject who received chronic dialysis, we randomly selected one subject who received conservative care based on nearest neighbor matching without replacement using calipers of width equal to 0.1 standard deviation of the logit of the propensity score. Comparisons of mortality rates and healthcare costs between groups were performed. Microsoft SQL Server 2012 (Microsoft Corporation, Redmond, WA, USA) was used for data linkage, processing and sampling. Propensity score was calculated using SAS software (version 12.0; SAS Institute, Inc., Cary, NC, USA). All other statistical analyses were conducted using STATA statistical software (version 12.0; StataCorp., College Station, Texas, USA). Statistical significance was defined as *P* <0.05.

## Results

### Characteristics of the study population

We identified 8,341 older patients with advanced CKD (estimated GFR <15 ml/min/1.73 m^2^) who met the inclusion criteria between January 2000 and December 2010. The mean age was 79.4 (standard deviation (SD) 7.0) years. Most (55.3%) patients were female, and the mean CCI was 5.6 (SD 2.1). A plurality of patients had the following comorbidities: hypertension, diabetes mellitus or dyslipidemia. Common concomitant medications were calcium channel blockers, diuretics, ACEIs or ARBs and dipyridamole, followed by antiplatelet agents (Table 1). During the follow-up period, 75.4% of the study subjects (n = 6,292) received chronic dialysis, and the rest (n = 2,049) received conservative care.

### Incidence rate and risk of mortality among older patients with advanced CKD

**Figure 1:**
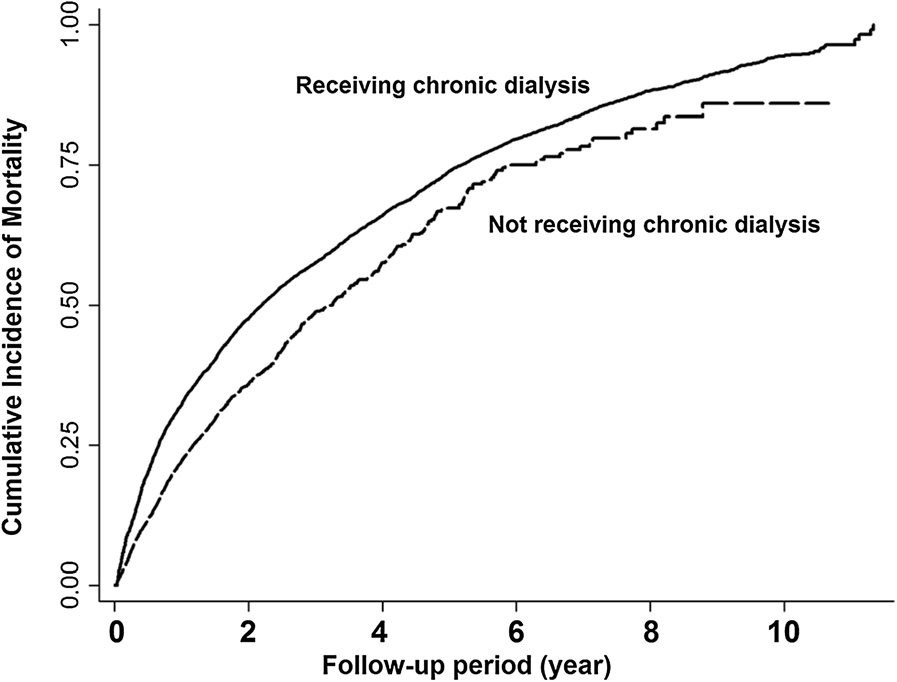
**Cumulative incidence of mortality among older patients with advanced chronic kidney disease.**

A total of 5,807 deaths occurred in older patients with advanced CKD during the follow-up period of 28,397 person-years. The overall mortality rate was 204.5 per 1,000 person-years. Compared with conservative care, chronic dialysis was associated significantly with a higher mortality risk (adjusted HR (aHR) 1.39, 95% CI 1.30 to 1.49, *P* <0.001; Table 2). The cumulative incidence of mortality is illustrated in Figure 1. Other significant risk factors for mortality among older patients with advanced CKD included older age (aHR per age 1.07, 95% CI 1.06 to 1.07), male (aHR 1.11, 95% CI 1.05 to 1.17), higher CCI (aHR per score 1.08, 95% CI 1.07 to 1.10), parkinsonism (aHR 1.24, 95% CI 1.13 to 1.37), use of diuretic (aHR 1.14, 95% CI 1.07 to 1.20) or steroid (aHR 1.11, 95% CI 1.01 to 1.21) (Table 3).

**Table 2:** **Hazard ratios for mortality according to receipt of chronic dialysis during follow up**

**Variables**	**Number of deaths**	**Exposure time, patient-years**	**Crude hazard ratio (95%** **CI)**	***P*** **value**	**Adjusted hazard ratio** ^**a**^ **(95%** **CI)**	***P*** **value**
Not receiving chronic dialysis	1,699	13,071	(Referent)		(Referent)	
Receiving chronic dialysis^b^	4,108	15,326	1.35 (1.26 to 1.44)	<0.001	1.39 (1.30 to 1.49)	<0.001

^a^Adjusted for all covariates listed in Table 1. ^b^Chronic dialysis was calculated as a discrete time-dependent covariate. CI, confidence interval.

**Table 3:** **Significant risk factors for mortality among older patients with advanced chronic kidney disease**

	**Adjusted** ^**a**^	
**Variables**	**Hazard ratio (95%** **CI)**	***P*** **value**
Age, per year	1.07 (1.06 to 1.07)	<0.001
Male	1.11 (1.05 to 1.17)	<0.001
Charlson comorbidity index score, per 1 point	1.08 (1.07 to 1.10)	<0.001
Receiving chronic dialysis^b^	1.39 (1.30 to 1.49)	<0.001
Dyslipidemia	0.86 (0.81 to 0.91)	<0.001
Parkinsonism	1.24 (1.13 to 1.37)	<0.001
Calcium channel blocker use	0.88 (0.83 to 0.93)	<0.001
Diuretic use	1.14 (1.07 to 1.20)	<0.001
Dipyridamole use	0.93 (0.88 to 0.99)	0.019
Steroid use	1.11 (1.01 to 1.21)	0.034

^a^Adjusted for all ovariates in Table 1. ^b^Chronic dialysis was calculated as a discrete time-dependent covariate. CI, confidence interval.

**Figure 2:**
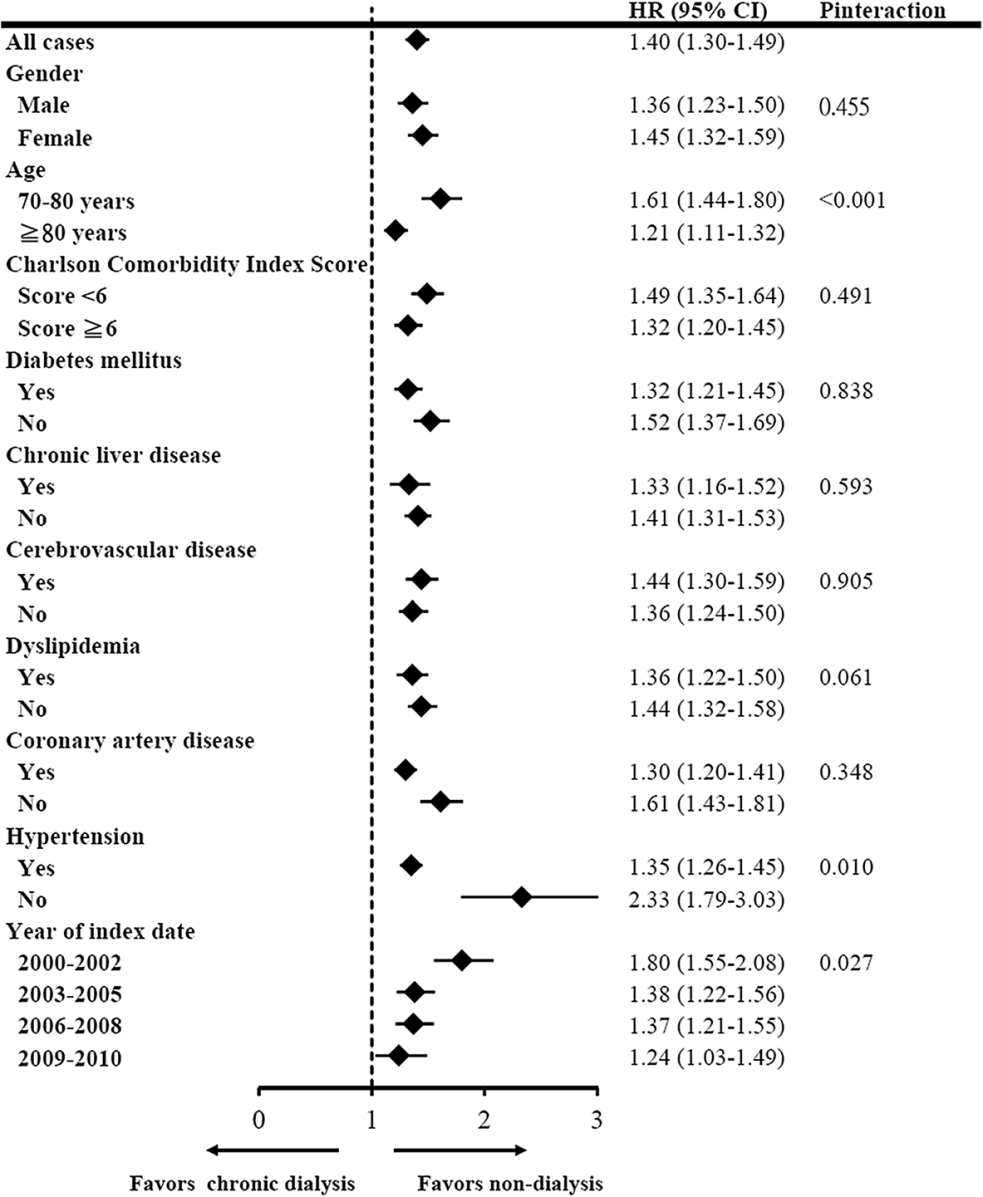
**Subgroup analyses of associations between chronic dialysis and mortality risk among older patients with advanced chronic kidney disease.**

In subgroup analysis, age (*P* <0.001), hypertension (*P* = 0.010) and year of index date (*P* = 0.027) interacted significantly with the receipt of chronic dialysis. Compared with conservative care, chronic dialysis increased mortality risk among patients <80 years old, those without hypertension and those diagnosed with advanced CKD between 2000 and 2002 (Figure 2).

### Healthcare expense in older patients with advanced CKD undergoing chronic dialysis versus conservative care

During the 28,397-person-year follow-up period, chronic dialysis was initiated for 6,292 patients at a median of 192 days after enrollment and a mean age of 79.4 (SD 7.1) years. Total healthcare costs, including total hospital and ambulatory visit costs, were included in our analysis. Healthcare utilization costs were retrieved primarily from NHI records, and also from insurance deductible data. Total healthcare cost categories were dialysis costs, laboratory and examination fees, medication costs, fees for procedures or surgery and nurse and physician fees. The mean healthcare cost for patients receiving chronic dialysis was US$23,994 (95% CI $23,616 to $24,371) per person-year; this cost was US$8,738 (95% CI $8,409 to $9,068) per person-year for the pre-dialysis period and US$42,980 (95% CI $37,050 to $48,909) per person-year for the period after initiation of chronic dialysis. The mean healthcare cost among the 2,049 patients who never received chronic dialysis was US$18,252 (95% CI $16,990 to $19,514) per person-year (Table 4).

**Table 4:** **Per-person and total costs attributable to advanced chronic kidney disease**

**Parameters**	**All patients**	**Not receiving chronic dialysis**	**Receiving chronic dialysis during follow-up period**
Number of patients	8,341	2,049	6,292
Age at time of enrollment, mean (SD), years	79.4 (7.0)	82.0 (6.4)	78.6 (7.1)
Total follow-up period, median (IQR), days	759 (302 to 1537)	279 (124 to 613)	985 (475 to 1758)
Day of chronic dialysis initiation, median (IQR)	-	-	192 (90 to 404)
Age at chronic dialysis initiation, mean (SD), years	-	-	79.4 (7.1)
Costs during total follow-up period, $US/person-years (95% CI)			
Total healthcare cost	22,583 (22,159 to 23,007)	18,252 (16,990 to 19,514)	23,994 (23,616 to 24,371)
Total hospital cost	10,980 (10,553 to 11,407)	14,448 (13,230 to 15,665)	9,850 (9,450 to 10,251)
Total ambulatory visit cost	11,603 (11,444 to 11,763)	3,805 (3,646 to 3,963)	14,143 (13,982 to 14,305)
Costs before chronic dialysis, $US/person-years (95% CI)			
Total healthcare cost	-	-	8,738 (8,409 to 9,068)
Total hospital cost	-	-	4,450 (4,158 to 4,743)
Total ambulatory visit cost	-	-	4,287 (4,199 to 4,377)
Costs after chronic dialysis initiation, $US/person-years (95% CI)			
Total healthcare cost	-	-	42,980 (37,050 to 48,909)
Total hospital cost	-	-	24,333 (18,460 to 30,208)
Total ambulatory visit cost	-	-	18,646 (18,309 to 18,984)

CI, confidence interval; IQR, interquartile range; SD, standard deviation.

### Propensity score–matched analysis

To further validate the study results, a propensity score analysis including 1,984 study subjects who received chronic dialysis and 1,984 matched patients who did not receive chronic dialysis was conducted. Potentially confounding baseline characteristics did not differ significantly between these groups [see Additional file 1: Table S1]. Compared with matched subjects who received conservative care, patients who received chronic dialysis had a significantly increased risk of mortality (HR 1.16, 95% CI 1.07 to 1.25, *P* <0.001; Additional file 1: Table S2) and higher total healthcare cost (US$26,273 *versus* US$17,973 per person-year; *P* <0.001; Additional file 1: Table S3).

## Discussion

This nationwide population-based study provides novel evidence that dialysis therapy does not always provide a substantial survival advantage among older patients with advanced CKD. Most of these patients face the prospect of dialysis therapy for the remainder of their lives. Thus, an understanding of the survival benefit of dialysis for this population is important. Using the initiation of chronic dialysis as a time-dependent variable in a Cox regression model, we found that dialysis therapy was associated with a nearly 40% increase in mortality risk, in patients ≥70 years old compared with those receiving conservative care. These increases remained significant in propensity score-matched analysis.

The risk of approaching dialysis was found to exceed that of mortality in most older patients with estimated GFR <15 ml/min/1.73 m^2^ [26]. Some authors have argued that older patients may have no real choice in dialysis decision making due to the lack of comprehensive insurance coverage, which may prohibit access to dialysis through implicit or explicit dialysis rationing due to limited medical resources and financial barriers [27-29]. Our study results, however, suggest that NHI coverage of dialysis expenses eliminates financial barriers for patients who may benefit from the treatment. Furthermore, nephrologists in Taiwan cannot legally withdraw or withhold dialysis without patient agreement, even when they believe that the treatment will have no benefit or that any benefit is outweighed by the burdens of treatment. In other words, patients and their families take active roles in dialysis decision making. Thus, our findings provide unrestricted objective evidence that can be used to optimize the risk-benefit analysis of dialysis therapy in older patients with advanced CKD.

The survival of patients undergoing dialysis in Taiwan improved rapidly after the initiation of the NHI program in 1995. However, outcomes of older patients have not improved substantially despite public insurance benefits enabling free healthcare access and total coverage of medical expenses [30]. Furthermore, Wu and colleagues [31] found that incident dialysis has been associated with a 6.27- to 10.4-fold greater risk of mortality in patients ≥70 years old compared with those <30 years old, even after adjusting for CCI. Among US nursing home residents, initiation of dialysis was related to substantial functional decline and up to 60% mortality within one year [32]. The cost of dialysis care also increases with age and the number of comorbidities [33]. In our study, the average annual per-patient cost of dialysis was much higher than that of conservative care among older patients; dialysis increased the total cost by US$5,742 per patient year. The healthcare cost was also significantly higher after initiation of chronic dialysis than in the pre-dialysis period (US$42,980 *versus* US$8,738 per patient year). In 2011, Medicare expenditures for dialysis in the US were $71,630 to $87,945 per patient year [1], at least double the costs calculated in the present study. Thus, ample opportunity exists to improve the outcomes of older patients after the initiation of dialysis therapy from a global standpoint. Alternatively, our findings support that robust conservative care was encouraged when considering opportunity cost principles. However, risk-benefit analysis in the present study is insufficient for decision making about dialysis for older patients; examination of this issue requires further long-term assessment.

Most previous small-scale studies [10-12,34,35] found that dialysis therapy conferred a modest (2 to 45.9 months) absolute survival advantage over conservative care in older patients, although this advantage was largely offset by high comorbidity rates. By contrast, our study demonstrated that dialysis therapy in older patients increased mortality risk by almost 40% compared with conservative care. This discrepancy may be due to differences in enrollment criteria and drawbacks of the statistical methods used in previous studies, such as the consideration of initiation of chronic dialysis as a dichotomous (non–time-dependent) variable in Cox proportional hazards models. This approach involves the examination of the relationship between survival and patient characteristics at the time of study enrollment, which may result in a false increase in survival rate in the dialysis group because patients who survived longer had an increased likelihood of receiving dialysis therapy. Several prognostic models have been developed to resolve this issue [36-38]. The definition of initiation of chronic dialysis as a time-dependent covariate in the present study allowed for the divergence of survival curves after this event had occurred. This type of model ensures that the risk of mortality increases only after dialysis initiation, which is more accurate and clinically relevant.

Our subgroup analyses showed that the risk of mortality was consistently elevated in older patients receiving dialysis therapy, regardless of age, sex, comorbidities or time cohort. Mortality risk was lower in patients >80 years old than in those 70 to 80 years old, implying that the impact of dialysis on increased mortality risk became less dominant with advanced age in patients with advanced CKD. The influence of dialysis on mortality in very old patients may be offset by competition between the risks of dialysis and death. The mortality risk associated with dialysis increased consistently, irrespective of specific comorbidities; however, the adjusted HR of mortality was lower in patients with, than in those without, hypertension. This result may be due to the vulnerability of older patients receiving dialysis to intradialytic hypotension, such that those with higher blood pressure targets may have a survival benefit [39]. Moreover, the effect of dialysis on mortality risk was smaller in the 2009 to 2010 cohort than in the 2000 to 2002 cohort. This finding may be attributed to advances in medical therapy and improvement in dialysis care over time.

Our study has several strengths. First, we present the largest currently available database for older patients with advanced CKD and their dialysis outcomes, with an extended follow-up period. Second, previous studies found that late or no referral to a nephrologist may be associated not only with an increased risk of short-term mortality, but also with incomplete understanding of dialysis in decision making [8,34]. Subjects in our study were referred early to nephrologists, with a median interval of 192 days between enrollment and dialysis. This characteristic eliminated selection bias because patients were placed under nephrologists’ care with the intent of initiating dialysis.

Our results provide objective information to facilitate dialysis decision making among older patients, their family members and physicians; however, some limitations of this study should be acknowledged. First, decisions about whether to receive dialysis may depend on patient preferences (family support, financial constraints and the will to live), but examination of this complex process was beyond the scope of the current study. Second, information on several potential confounding factors, including obesity, nutritional condition, psychosocial function, performance status and indication for dialysis initiation, were not available in the NHIRD database. Estimated GFRs at dialysis initiation were also not recorded, whereas the Initiating Dialysis Early and Late (IDEAL) Study showed no significant survival difference between early initiation (estimated GFRs: 10.0 to 14.0 ml per minute) and late initiation (estimated GFRs: 5.0 to 7.0 ml per minute) of dialysis [40]. Third, our study was subject to the inherent limitations of its retrospective and observational design. However, randomized control trials investigating this issue are not possible because of the potential of violating medical ethics. Finally, we based the diagnosis of advanced CKD on ESA prescriptions, resulting in the exclusion of patients with advanced CKD who never received such prescriptions (that is, those who had no obvious renal anemia). The results of the study cannot be generalized to all older patients with advanced CKD.

## Conclusions

Our findings provide novel information useful for the reconsideration of dialysis decision making that accounts for risk-benefit relationships in older patients with advanced CKD. For frail older patients with late-stage CKD, conservative care may be a viable treatment option, as dialysis may not prolong life expectancy.

## Additional file

Additional file 1: Table S1. Baseline characteristics of patients after propensity-score matching. **Table S2.** Crude and adjusted hazard ratios for mortality according to receiving chronic dialysis during the follow-up period. **Table S3.** Per person and total costs attributable to advanced chronic kidney disease.
