# Supplementary material for: The impact of dialysis therapy on older patients with advanced chronic kidney disease: a nationwide population-based study
Source: BMC Med. 2014 Oct 6;12:169. doi: 10.1186/s12916-014-0169-3 (PMC4189680; doi:10.1186/s12916-014-0169-3)
Supplement: Additional file 1: Table S1. — Baseline characteristics of patients after propensity-score matching. Table S2. Crude and adjusted hazard ratios for mortality according to receiving chronic dialysis during the follow-up period. Table S3. Per person and total costs attributable to advanced chronic kidney disease. [file 12916_2014_169_MOESM1_ESM.docx]

| **Supplementary Table 1. Baseline Characteristics of Patients After Propensity-Score Matching** | | | | |
| --- | --- | --- | --- | --- |
|  |  | **Propensity-score matched** | | |
|  |  | **Not receiving chronic dialysis** | **Receiving chronic dialysis** | **Standardized Difference ^d^** |
| **No. of patients** |  | 1,984 | 1,984 |  |
| **Male, *n* (%)** |  | 918 (46.2%) | 916 (46.1%) | -0.002 |
| **Age, mean (SD), year** |  | 81.6 (6.1) | 81.3 (6.1) | -0.045 |
| **Monthly income (US$), *n* (%)** |  |  |  |  |
| **Dependent** |  | 798 (40.2%) | 819 (41.2%) | 0.022 |
| **0-637** |  | 486 (24.4%) | 481 (24.2%) | -0.006 |
| **637-1,400** |  | 690 (34.7%) | 670 (33.7%) | -0.021 |
| **> 1,400** |  | 10 (0.5%) | 14 (0.7%) | 0.026 |
| **Urbanization^a^, *n* (%)** |  |  |  |  |
| **1** |  | 988 (49.7%) | 966 (48.6%) | -0.022 |
| **2** |  | 776 (39.1%) | 780 (39.3%) | 0.004 |
| **3** |  | 174 (8.7%) | 192 (9.6%) | 0.031 |
| **4** |  | 46 (2.3%) | 46 (2.3%) | 0.000 |
| **Primary renal disease** |  |  |  |  |
| **Diabetes** |  | 707 (35.6%) | 724 (36.4%) | 0.018 |
| **Glomerulonephritis** |  | 946 (47.6%) | 968 (48.7%) | 0.026 |
| **Secondary glomerulonephritis/Vasculitis** |  | 25 (1.2%) | 22 (1.1%) | -0.012 |
| **Hypertension/Large vessel disease** |  | 262 (13.2%) | 235 (11.8%) | -0.048 |
| **Cystic/Hereditary/Congenital disease** |  | 139 (7%) | 135 (6.8%) | -0.008 |
| **Miscellaneous conditions** |  | 53 (2.6%) | 50 (2.5%) | -0.011 |
| **Charlson comorbidity index score**^c^**, (SD)** |  |  |  | -0.014 |
| **Comorbid disease, *n* (%)** |  |  |  |  |
| **Diabetes mellitus** |  | 1,127 (56.8%) | 1,120 (56.4%) | -0.007 |
| **Hypertension** |  | 1,851 (93.2%) | 1,824 (91.9%) | -0.052 |
| **Dyslipidemia** |  | 878 (44.2%) | 807 (40.6%) | -0.072 |
| **Atrial fibrillation** |  | 161 (8.1%) | 149 (7.5%) | -0.023 |
| **Valvular heart disease** |  | 457 (23%) | 433 (21.8%) | -0.029 |
| **Parkinsonism** |  | 179 (9%) | 208 (10.4%) | 0.049 |
| **Autoimmue disease** |  | 298 (15%) | 266 (13.4%) | -0.046 |
| **Drug abuse** |  | 13 (0.6%) | 13 (0.6%) | 0.000 |
| **Concomitant medications, *n* (%)** |  |  |  |  |
| **Antiplatelet agents^a^** |  | 497 (25%) | 477 (24%) | -0.023 |
| **Warfarin** |  | 10 (0.5%) | 9 (0.4%) | -0.007 |
| **ACE inhibitors or ARB** |  | 660 (33.2%) | 636 (32%) | -0.026 |
| **Beta Blockers** |  | 82 (4.1%) | 84 (4.2%) | 0.005 |
| **Calcium channel Blockers** |  | 968 (48.7%) | 944 (47.5%) | -0.024 |
| **Diuretics** |  | 1,074 (54.1%) | 1,093 (55%) | 0.019 |
| **Nitrate** |  | 368 (18.5%) | 356 (17.9%) | -0.016 |
| **Statins** |  | 143 (7.2%) | 126 (6.3%) | -0.034 |
| **Dipyridamole** |  | 457 (23%) | 451 (22.7%) | -0.007 |
| **Steroid** |  | 184 (9.2%) | 188 (9.4%) | 0.007 |
| **Estrogen or progesterone** |  | 11 (0.5%) | 9 (0.4%) | -0.014 |
| **Non-steroidal anti-inflammatory drugs** |  | 304 (15.3%) | 297 (14.9%) | -0.010 |
| **Selective serotonin re-uptake inhibitors** |  | 27 (1.3%) | 26 (1.3%) | -0.004 |
| **Proton-pump inhibitors** |  | 142 (7.1%) | 140 (7%) | -0.004 |
| **Oral hypoglycemic drugs** |  | 408 (20.5%) | 397 (20%) | -0.014 |
| *Abbreviations:* SD, standard deviation; ACE, angiotensin-converting enzyme; ARB, angiotensin II receptor blocker  **^a^** Urbanization levels in Taiwan are divided into four strata according to the Taiwan National Health Research Institute publications. Level 1 designates the most urbanized areas, and level 4 designates the least urbanized areas.  ^b^ Charlson Comorbidity Index (CCI) score is used to determine overall systemic health. With each increased level of CCI score, there are stepwise increases in the cumulative mortality[_._](#_ENREF_9)  ^c^ Including aspirin, clopidogrel, ticlopidine and cilostazol.  ^d^ Standardized difference = difference in means or proportions divided by standard error; imbalance  defined as absolute value greater than 0.062. | | | | |

| **Supplementary Table 2. Crude and adjusted hazard ratios for mortality according to receiving chronic dialysis during the follow-up period.** | | | | | |
| --- | --- | --- | --- | --- | --- |
|  | **All patients** | |  | **Propensity-score Matched** | |
|  | **No. of Mortality** | **Exposure time,**  **patient-years** |  | **Hazard ratio**  **(95% CI)** | **P value** |
| **Not receiving chronic dialysis** | 1,651 | 4,157 |  | (Referent) |  |
| **Receiving chronic dialysis ^b^** | 1,661 | 3,939 |  | 1.16 (1.07-1.25) | <0.001 |
| ^a^ Adjusted all covariates in Table 1.  ^b^ Chronic dialysis was calculated as a discrete time-varying covariate. | | | | | |

| **Supplementary Table 3. Per person and total costs attributable to Advanced chronic kidney disease** | | | | |
| --- | --- | --- | --- | --- |
|  | **Propensity-Score matched** | | | |
|  | **Not receiving chronic dialysis** |  | **Receiving chronic dialysis during follow-up period** | **P Value** |
| **Patient No.** | 1,984 |  | 1,984 |  |
| **Age on enrolment, mean (SD), year** | 81.6 (6.1) |  | 81.3 (6.1) | 0.153 |
| **Total Medicare cost, US$ Per Person-year (95% CI)** | 17,973 (16,742-19,205) |  | 26,273 (25,458-27,089) | <0.001 |
| **Total hospital cost, US$ Per Person-year (95% CI)** | 14,172 (12,987-15,356) |  | 13,194 (12,330-14,057) | 0.191 |
| **Total ambulatory visit cost, US$ Per Person-year (95% CI)** | 3,802 (3,642-3,961) |  | 13,080 (12,786-13,374) | <0.001 |
| *Abbreviations:* SD, standard deviation; US$, United States Dollars; CI confidence interval. | | | | |
